# Quality of life following lobectomy versus total thyroidectomy is significantly related to hypothyroidism

**DOI:** 10.1002/jso.26983

**Published:** 2022-06-11

**Authors:** Dan Yaniv, Igor Vainer, Ido Amir, Eyal Robenshtok, Dania Hirsch, Torquil Watt, Ohad Hilly, Yotam Shkedy, Thomas Shpitzer, Gideon Bachar, Raphael Feinmesser, Aviram Mizrachi

**Affiliations:** ^1^ Department of Otorhinolaryngology—Head and Neck Surgery Rabin Medical Center Petah Tikva Israel; ^2^ Sackler Faculty of Medicine Tel Aviv University Tel Aviv Israel; ^3^ Institute of Endocrinology, Diabetes and Metabolism Rabin Medical Center Petah Tikva Israel; ^4^ Department of Endocrinology National University Hospital Copenhagen Denmark; ^5^ Department of Otorhinolaryngology—Head and Neck Surgery, Rambam Healthcare Campus, Technion‐Israel Institute of Technology Rappaport Faculty of Medicine Haifa Israel

**Keywords:** hypothyroidism, PRO, QOL, thyroidectomy, TSH

## Abstract

**Objective:**

The aim of the present study was to investigate the differences in quality of life (QOL) following complete or partial thyroidectomy and with regard to thyroid hormone replacement (LT4) therapy.

**Study Design:**

Patients who underwent thyroidectomy were asked to complete the validated thyroid‐specific ThyPRO QOL questionnaire at least 6 months following surgery.

**Setting:**

Tertiary medical center.

**Methods:**

Thyroid specific QOL questionnaire analysis.

**Results:**

A total of 190 patients completed the ThyPRO questionnaire. Of them 89 patients had complete thyroidectomy and 101 patients had unilateral thyroid lobectomy. The total thyroidectomy group had significantly worse overall QOL self‐assessment score than the lobectomy patients (*p* < 0.0001). Patients receiving LT4 therapy regardless of the extent of surgery, reported worse QOL compared to patients not receiving LT4.

**Conclusions:**

Quality of life following thyroid surgery is significantly related to hypothyroidism and the requirement for LT4 therapy, rather to the extent of surgery. The best QOL was reported in patients treated with lobectomy who did not require LT4 therapy.

## 1 INTRODUCTION

The American Thyroid Association (ATA) guidelines^1^ state that either complete thyroidectomy or lobectomy is an acceptable surgical approach for patients with well differentiated thyroid cancer (WDTC) measuring 1−4 cm without evidence of extrathyroidal extension or clinically apparent lymph node involvement on preoperative evaluation. This is a strong recommendation with moderate‐quality evidence from large database studies, which reflects a trend toward less aggressive surgical approach in recent years.

Complications of thyroid surgery are not uncommon,^2^ so the advantages of limited surgery, including lower risk for recurrent laryngeal nerve injury and hypoparathyroidism, are important. Additionally, there is an increasing awareness that imperfections of thyroid replacement therapy have effects on general well‐being.^3^ Unfortunately, there are no randomized trials that compare the effectiveness of total thyroidectomy with a less extensive operation. Clinicians are left to choose the extent of thyroidectomy based on expert's opinion and evidence from retrospective studies.^4^

Health‐related quality of life is a major concern for patients with WDTC given the excellent prognosis of this disease. In comparison with the general population, quality of life (QOL) issues have been found in WDTC patients in areas such as insomnia, fatigue, and limitations of daily functioning.^5^, ^6^ Previous studies on quality of life in patients with WDTC were hampered by small sample size and limited quality of life parameters or were uncontrolled. The effect of hormone replacement therapy was not fully assessed in previous studies.

A study by Watt et al.^7^ supported the clinical validity of the new thyroid‐specific QOL questionnaire, ThyPRO, and reported good test−retest reliability. The questionnaire had been used in multiple clinical studies of patients with thyroid diseases. The aim of the present study was to thoroughly assess and compare QOL parameters in patients following partial and total thyroidectomy. Furthermore, we sought to investigate the impact of LT4 therapy on QOL in patients with iatrogenic hypothyroidism.

## 2 METHODS

### 2.1 Patients

Patients who underwent thyroidectomy at a university‐affiliated tertiary medical center and were at least 6 months following surgery were offered to participate in the study during routine clinic visits. After obtaining an informed consent, the patients were asked to independently complete the thyroid‐specific ThyPRO QOL questionnaire.

Questionnaire results were entered to an electronic database, and then verified by a second researcher to confirm that data entry was correct without errors.

In addition, data on patients' demographics, comorbidities, pathology, follow‐up, and thyroid function were collected from electronic medical records.

### 2.2 ThyPRO quality of life questionnaire

**Figure 1:**
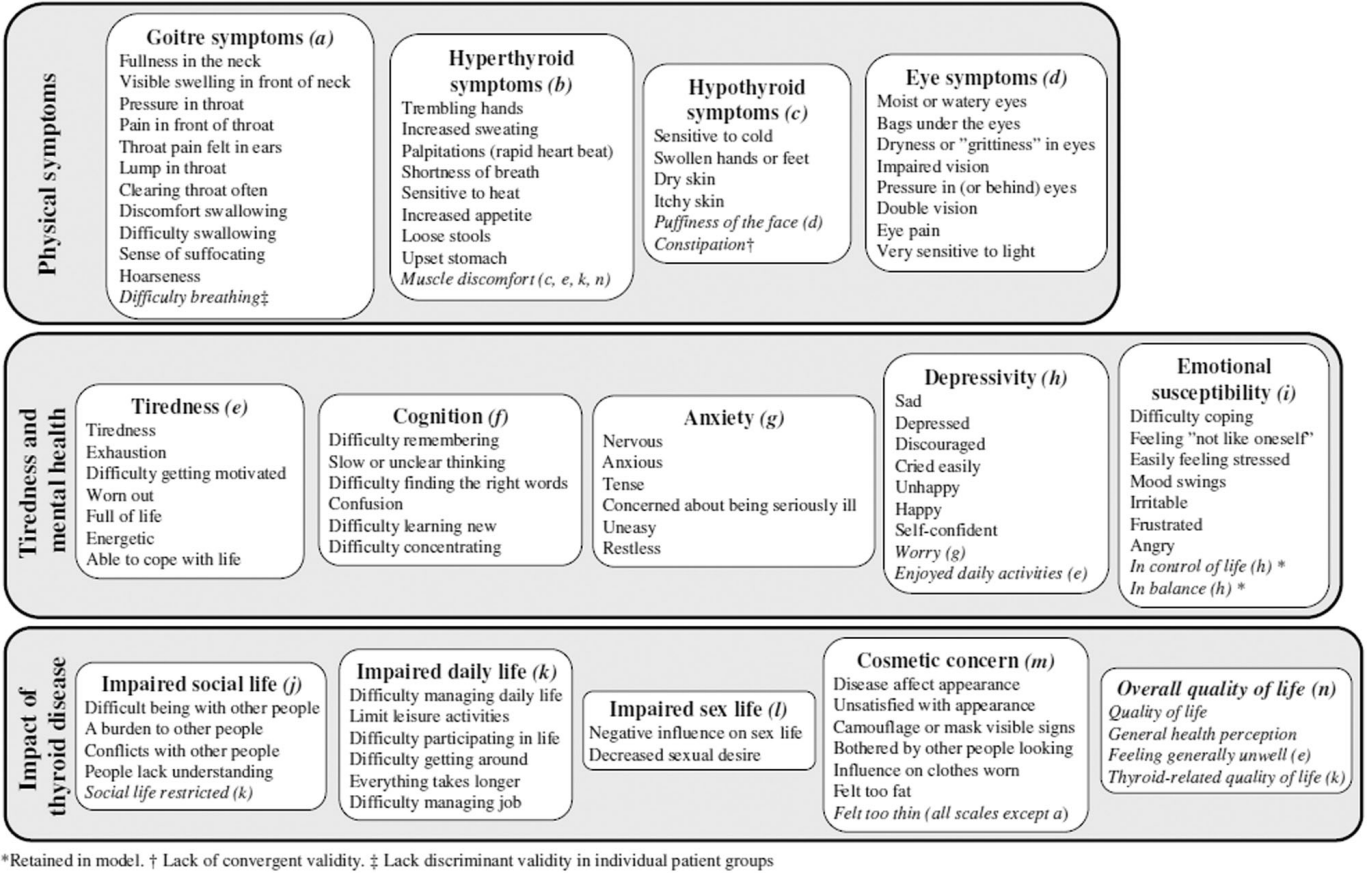
ThyPRO quality of life questionnaire three main categories and 14 subcategories

The ThyPRO quality of life questionnaire is a comprehensive validated patient‐reported outcomes tool that was designed to encompass all aspects of quality of life and everyday living. The questionnaire is composed of three main categories and 14 subcategories, each has between 2 and 12 questions, which sums up to a total of 85 questions per patient (Figure 1).

The first category relates to physical symptoms and includes different signs and symptoms associated with the neck region, thyroid function, and eyes. The second category relates to tiredness and mental health and includes different psychological and psychosomatic symptoms. The third category relates to impact on everyday life activities and includes social and interpersonal issues. The ThyPRO was introduced to the patients by the caregiver (surgeon or endocrinologist) during routine follow‐up visit and if the patient agreed to participate in the study, they received a coded unidentified questionnaire to complete in a secluded area. After completion, the questionnaires were collected by an independent research assistant who extracted the data into an electronic spreadsheet.

### 2.3 Statistical analysis

Data were analyzed using the SPSS statistical software version 25.0 (SPSS). Data distribution were analyzed using Shapiro−Wilk test to test whether the data derived from normal distribution.

For the analysis of continuous data Student's *t*‐test was used for normally distributed variables and Kruskal−Wallis for nonparametric variables. *χ* ^2^ or Fisher's test were utilized for analysis of categorical variables.

A two‐sided *p *< 0.05 was considered statistically significant. All presented means are accompanied by their respective standard deviations.

### 2.4 Ethical considerations

The study was approved by the institutional review board and informed consent was obtained from all individuals participating in the study. Rabin Medical Center IRB record #RMC‐0011‐18.

## 3 RESULTS

### 3.1 Clinical and demographic data

The study included 190 patients who completed the ThyPRO questionnaire. Patients with clinical or subclinical hypothyroidism based on TSH measurements (TSH > 4 μIU/ml) in proximity to the completion of the ThyPRO questionnaire, were excluded from the study, assuming QOL in these patients may be affected by hormonal imbalance.

**Figure 2:**
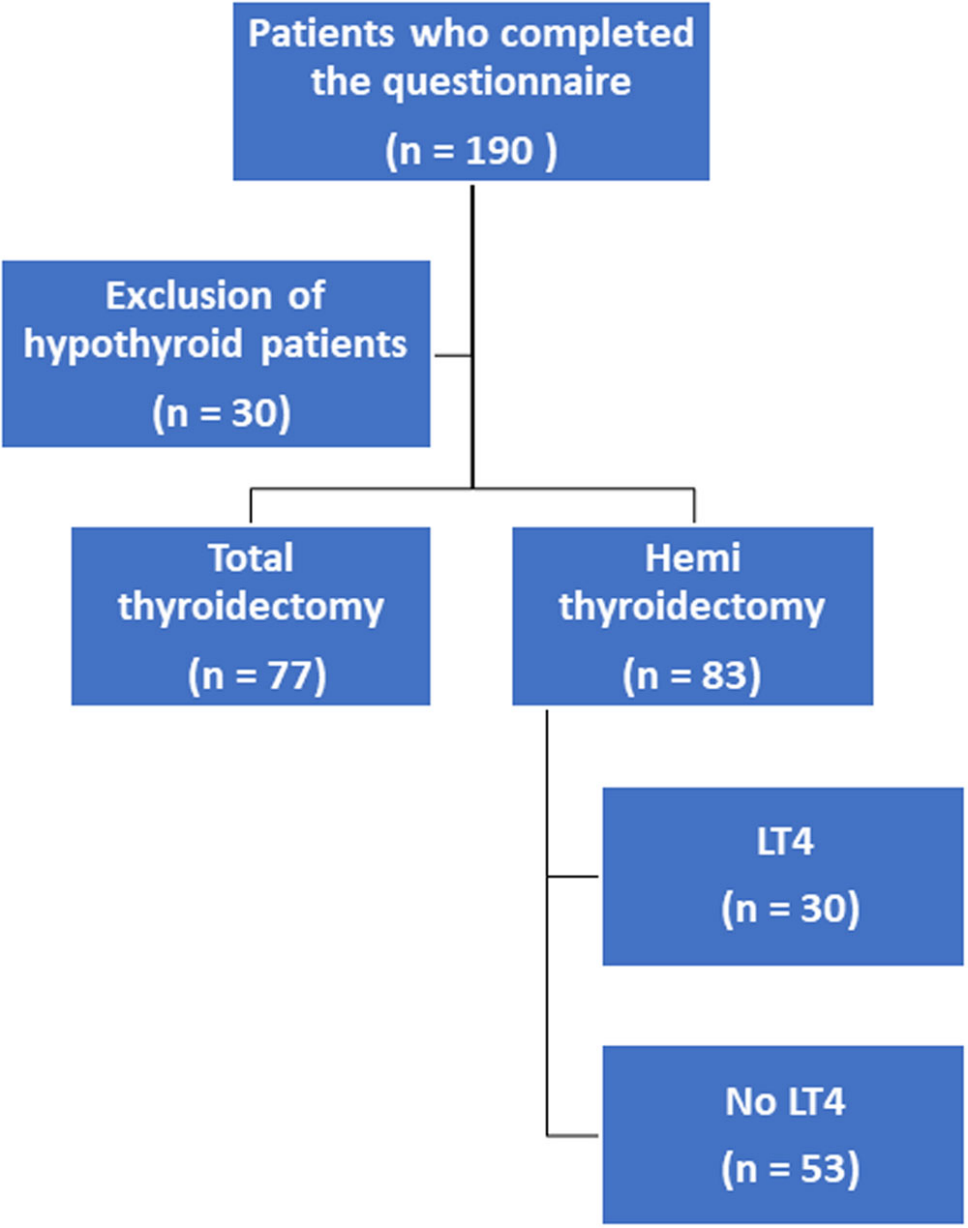
Distribution of 190 patients who completed the ThyPRO questionnaire

After exclusion, 160 patients with normal or suppressed TSH (as part of their treatment for thyroid cancer) were included in the final analysis, of which 77 patients had complete thyroidectomy and 83 patients unilateral thyroid lobectomy. Figure 2 illustrates the distribution of all patients who completed the ThyPRO questionnaire.

Mean age was 54 years (range: 22−88) and the majority of patients were females (133 patients, 83.1%). Papillary thyroid carcinoma (PTC) was the most common histology and was noted in 118 patients (73.8%) followed by benign multinodular goiter in 32 patients (20%), Hürthle cell adenoma in 4 patients (2.5%), and medullary thyroid carcinoma in 3 patients (1.8%). Clinical and demographic data are presented in Table 1.

**Table 1:** Clinical and demographic data of patients

Parameter	Mean/*N* (%)
Age (years)	54.5 (range: 22−88)
Gender—female	133 (83.1%)
Surgery type	
Hemithyroidectomy	83 (51.8%)
Total thyroidectomy	77 (48.2%)
Comorbidities	60 (37.5%)
Adjuvant RAI treatment	58 (36.3%)
Histology	
Papillary carcinoma	118 (73.8%)
Goiter	32 (20%)
Hürthle cell adenoma	4 (2.5%)
Medullary carcinoma	3 (1.85%)
Missing data	3 (1.85%)

Abbreviation: RAI, radioactive iodine.

### 3.2 Unilateral thyroid lobectomy versus total thyroidectomy

Comparison of clinical and pathological parameters as well as ThyPRO scores between patients who underwent unilateral thyroid lobectomy and total thyroidectomy is presented in Table 2.

**Table 2:** Comparison between total thyroidectomy and hemithyroidectomy patients

Parameter	Total thyroidectomy (*n* = 77)	Hemithyroidectomy (*n* = 83)	*p* Value
Age (years)	57.4 ± 15.7	51.1 ± 18.01	0.03
Gender—female	56 (72.7%)	77 (92.8%)	0.001
Comorbidities	31 (40.3%)	29 (34.9%)	0.44
Full LT4 treatment	73 (94.8%)	20 (24.1%)	<0.0001
Pathology			
Goiter	2 (2.6%)	30 (36.1%)	<0.0001
Hürthle cell adenoma	1 (1.3%)	3 (3.6%)	1
Papillary carcinoma	57 (93.4%)	43 (54.4%)	0.003
Medullary carcinoma	2 (2.6%)	1 (1.2%)	1
Missing data	0	3 (3.6%)	1
ThyPRO scores			
Goiter	15.93 ± 16.98	17.48 ± 18.11	0.576
Hyper symptoms	16.81 ± 15.56	13.93 ± 15.04	0.236
Hypo symptoms'	16.8 ± 19.14	18.45 ± 22.41	0.619
Eye symptoms	14 ± 14.54	10.13 ± 14.42	0.093
Tiredness	44.9 ± 21.1	37.87 ± 25.46	0.06
Cognitive	20.29 ± 23.84	17.42 ± 22.45	0.434
Anxiety	28.73 ± 27.78	24.95 ± 25.85	0.373
Depression	28.61 ± 20.58	24.53 ± 21.95	0.227
Emotional	34.49 ± 21.54	28.28 ± 22.83	0.079
Impaired social life	10.39 ± 18.57	8.13 ± 16.5	0.417
Impaired daily life	16.88 ± 23.06	15.88 ± 22.61	0.782
Impaired sex life	17.53 ± 27.67	10.69 ± 19.73	0.072
Cosmetic complaints	17.71 ± 26.19	12.7 ± 20.67	0.18
Self‐assessment	26.62 ± 36.12	8.87 ± 21.33	<0.0001

**Figure 3:**
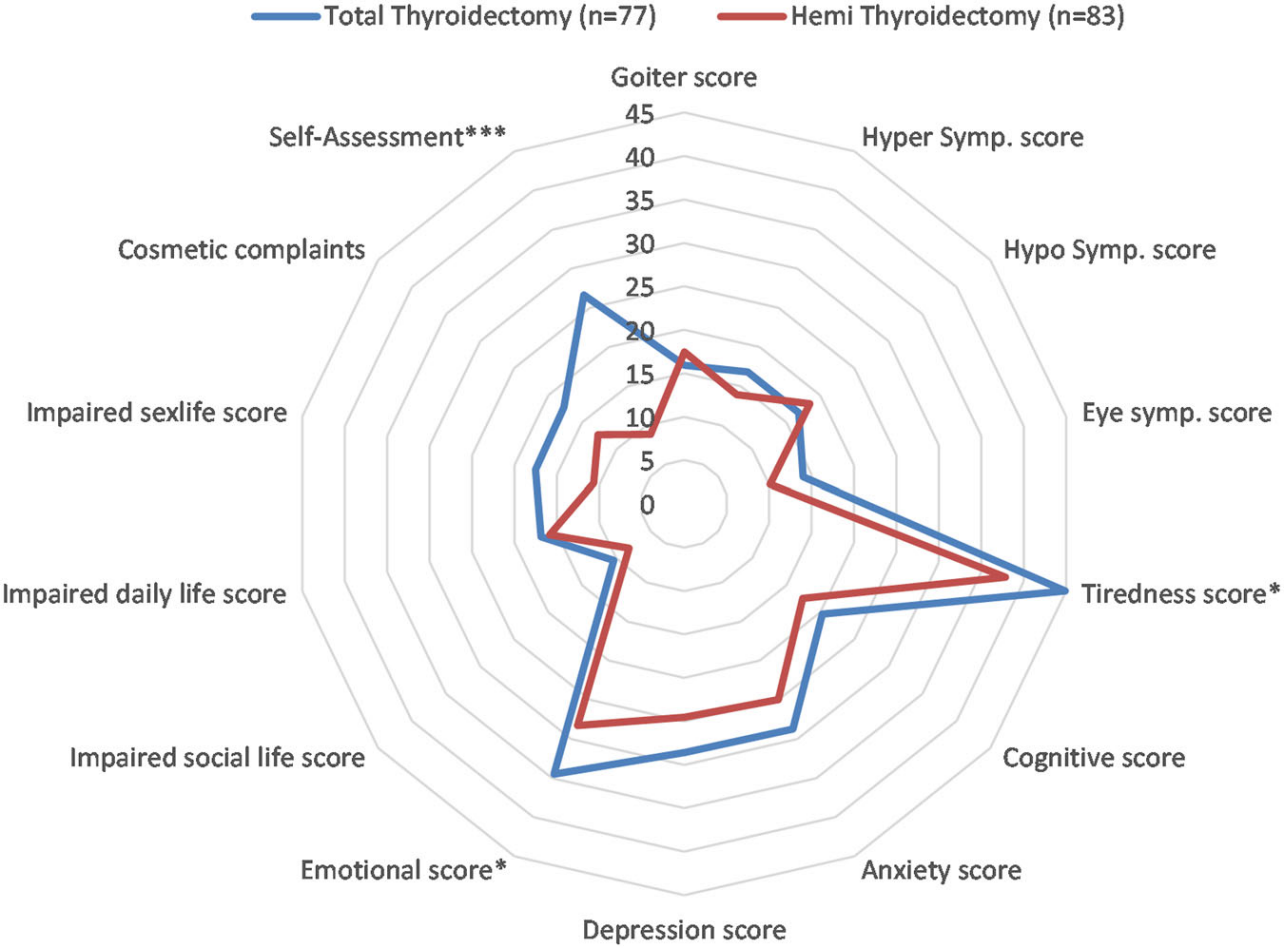
Comparison of ThyPRO parameters between thyroid lobectomy and total thyroidectomy patients (*p < 0.1, **p < 0.05, ***p < 0.01)

The results showed a statistically significant difference in the self‐assessment score. This score represents the overall negative impact of the thyroid disease on patient's QOL and was higher in the total thyroidectomy group (26.6 ± 36.1 vs. 8.9 ± 21.3, *p* < 0.0001). Furthermore, patients in the total thyroidectomy group reported higher tiredness, impaired sex‐life, and emotional scores compared to the unilateral thyroid lobectomy group. Other parameters such as hypothyroid score (16.8 ± 19.14 in total thyroidectomy group vs. 18.45 ± 22.41 in lobectomy group, *p* = 0.619), hyperthyroid score (16.81 ± 15.56 in total thyroidectomy group vs. 13.93 ± 15.04 in lobectomy group, *p* = 0.236), and cognitive score (20.29 ± 23.84 in total thyroidectomy group vs. 17.42 ± 22.45 in lobectomy group, *p* = 0.434) did not show a statistically different score between the groups (Figure 3).

For impaired sex life we also compared the young patient group (under 50 years of age) and the old patient group (over 50 years of age). After adjusting to age we found that in the younger age group (patients under 50 years of age) the score was 19.44 for total thyroidectomy group and 11.84 for the hemithyroidectomy group (*p* = 0.19), in the older patient group (patients over 50 years of age) the scores were 16.5 and 9.72, respectively (*p* = 0.161).

### 3.3 Patients receiving LT4 therapy versus patients not receiving LT4 therapy

Out of 160 patients, 107 (66.8%) were receiving LT4 replacement therapy.

**Figure 4:**
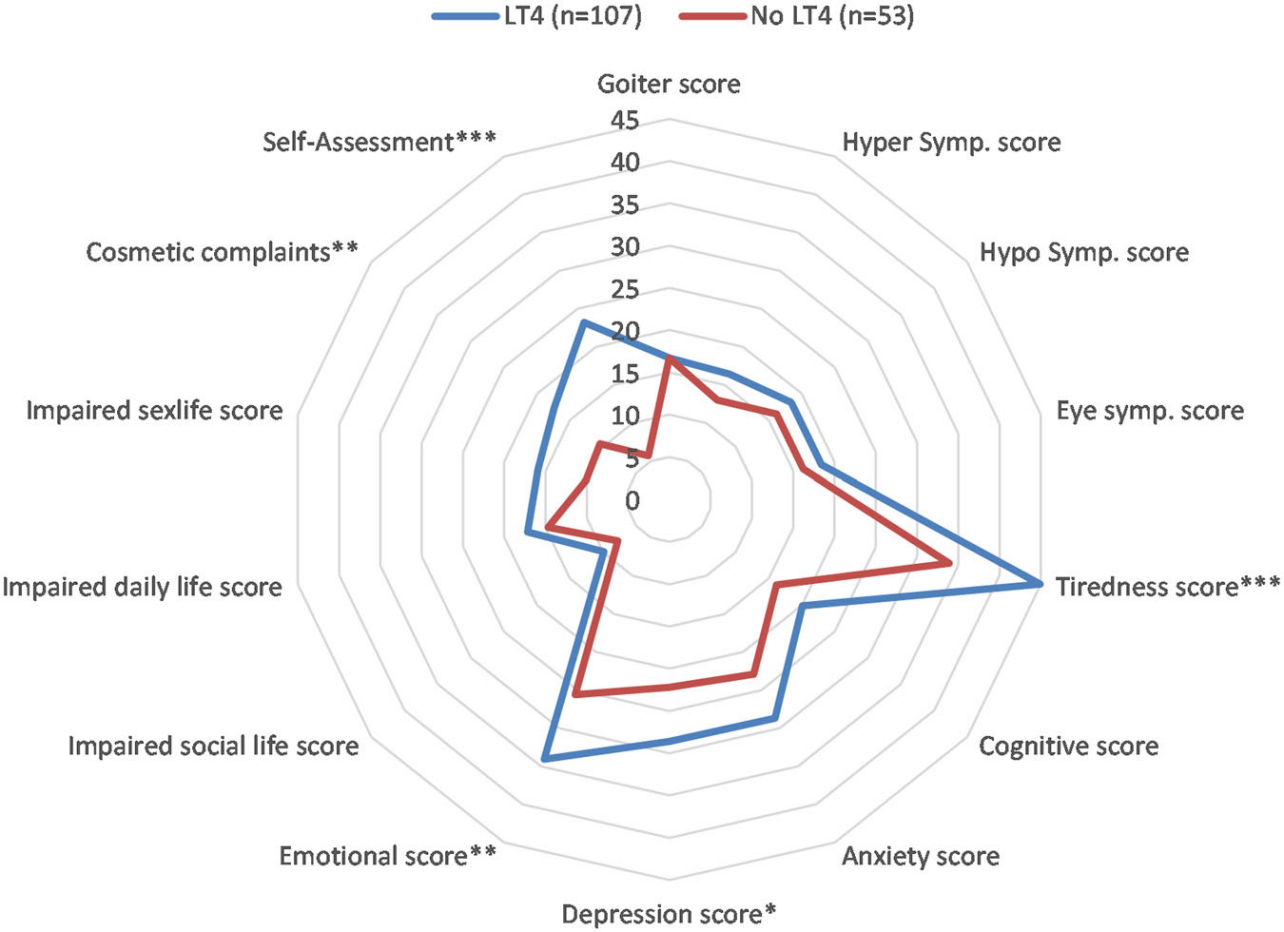
Comparison of ThyPRO parameters between patients who receive LT4 treatment and patients who do not receive LT4 treatment (*p < 0.1, **p < 0.05, ***p < 0.01)

Patients receiving LT4 therapy regardless of the extent of surgery had higher tiredness score (44.86 ± 22.07 vs. 33.96 ± 25.22, *p* = 0.006), emotional score (34.09 ± 21.87 vs. 25.57 ± 22.48, *p* = 0.02), cosmetic complaints (17.41 ± 26.07 vs. 10.45 ± 16.64, *p* = 0.04), and overall self‐assessment score (23.23 ± 33.87 vs. 5.66 ± 17.8, *p* = 0.001). In addition, depression score showed marginal statistical significance (*p* = 0.06), with higher score in the LT4 group (28.6 ± 21.5 vs. 22.2 ± 20.5) (Figure 4).

### 3.4 Lobectomy patients receiving LT4 therapy versus total thyroidectomy patients

Of the 83 patients who underwent unilateral thyroid lobectomy, 30 (36.1%) were receiving LT4 supplements/replacement therapy. These patients were compared to all 77 patients in the total thyroidectomy group. The overall self‐assessment score was higher in total thyroidectomy group (26.62 ± 36.12 vs. 14.53 ± 25.81, *p* = 0.07).

There were no statistically significant difference between the lobectomy patients receiving LT4 therapy and the total thyroidectomy patients in hyperthyroid score (16.81± 15.56 in total thyroidectomy group vs. 15.52 ± 15.88 in lobectomy group, *p* = 0.47), hypothyroid score (16.8 ± 19.13 in total thyroidectomy group vs. 22.5 ± 28.55 in lobectomy group, *p* = 0.22), cognition (20.29 ± 23.84 in total thyroidectomy group vs. 19.58 ± 24.25 in lobectomy group, *p* = 0.51), and impaired daily life (16.88 ± 23.06 in total thyroidectomy group vs. 17.91 ± 24.8 in lobectomy group, *p* = 0.54).

### 3.5 Lobectomy patients receiving LT4 therapy versus lobectomy patients without LT4 therapy

**Figure 5:**
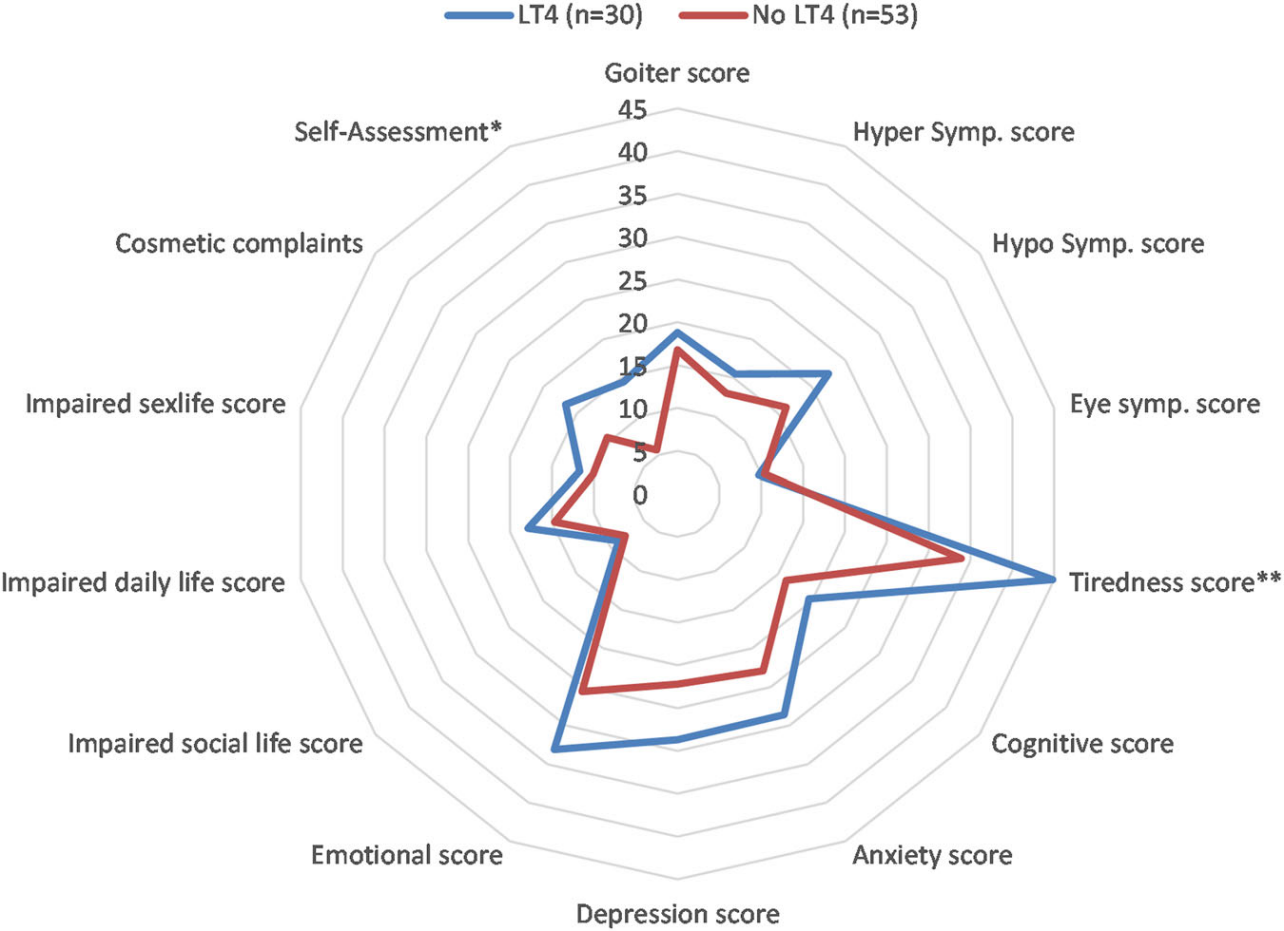
Comparison of ThyPRO parameters between lobectomy patients who receive LT4 treatment and lobectomy patients who do not receive LT4 treatment (*p < 0.1, **p < 0.05, ***p < 0.01)

Patients on LT4 therapy had a significantly higher tiredness score (44.76 ± 24.79 vs. 33.96 ± 25.22, *p* = 0.05) and higher overall self‐assessment score (14.53 ± 25.81 vs. 5.66 ± 17.08, *p* = 0.06). There were no difference between the groups in hyperthyroid score (15.52± 15.9 vs. 13.03 ± 14.62, *p* = 0.47), hypothyroid score (22.5 ± 28.55 vs. 16.15 ± 17.95, *p* = 0.22), cognition (19.58 ± 24.25 in the LT4 vs. 16.19 ± 21.51, *p* = 0.51), and impaired daily life (17.92 ± 24.81 vs. 14.73 ± 21.42, *p* = 0.54) (Figure 5).

### 3.6 Lobectomy patients receiving full LT4 therapy versus lobectomy patients on partial LT4 therapy

When looking at the lobectomy group receiving LT4 therapy, 15 patients (50%) received full replacement therapy, defined as a weekly dose of 80% or greater than the expected dose (1.7 mcg x body weight x 7) and 15 patients received only partial therapy (<80%).

No difference was found between the groups in hyperthyroid score (25.7 ± 28.38 in the full LT4 therapy group compared to the 11.7 ± 21.23 in the partial LT4 therapy group,*p* = 0.93), hypothyroid score (18.75 ± 26.34 vs. 28.13 ± 31.92, *p* = 0.37), tiredness score (39.48 ± 23.83 vs. 52.68 ± 25.07, *p* = 0.29), cognition (17.82 ± 25.64 vs. 22.23 ± 22.84, *p* = 0.52), anxiety (28.24 ± 25.27 vs. 29.17 ± 28.48, *p* = 0.98), depression (24.21 ± 20.45 vs. 35.41 ± 28.18, *p* = 0.37), and overall self‐assessment score (13.89 ± 23.04 vs. 15.5 ± 30.56, *p* = 0.47).

### 3.7 Patients with TSH suppression versus patients with TSH within normal range

We defined TSH suppression as patients receiving LT4 with TSH of less than 0.5 μIU/ml as per ATA guidelines for TSH suppression.^1^ Fifty‐eight patients had a suppressed TSH, compared to 102 patients with TSH within normal range. The overall self‐assessment score was higher in the suppressed TSH group compared to the normal TSH group (26.72 ± 35.93 vs 12.12 ± 25.87, *p* = 0.003), with no other scores showing statistically significant difference. 

### 3.8 Patients who received radioactive iodine (RAI) treatment versus patients who did not receive RAI treatment

We compared patients who received adjuvant RAI treatment following surgery to patients treated with surgery alone. In the total thyroidectomy group, 48 out of 77 (58.4%) patients received adjuvant RAI treatment following surgery. There were no statistically significant difference in any parameter between the patients who received RAI and those who did not.

### 3.9 Women before and after menopause

We compared between women over and under the age of 50 to assess whether menopause symptoms might be confounders in this patient population. The study included 77 (58%) women over the age of 50 and 56 (42%) women under 50.

No significant differences in QOL parameters were found between the two groups. Hyperthyroid score (16.08 ± 17.41in the over 50 group compared to 15.12 ± 13.32 in the younger group, *p* = 0.732), hypothyroid score (19.16 ± 22.84 vs. 19.87 ± 20.75, *p* = 0.854), tiredness score (41.19 ± 23.76 vs. 44.26 ± 25.23, *p* = 0.474), cognition (19.64 ± 25.27 vs. 19.35 ± 21.09, *p* = 0.943), depression (27.08 ± 19.97 vs. 29.02 ± 25.18, *p* = 0.622), and overall self‐assessment score (18.94 ± 31.13 vs. 16.09 ± 30.51, *p* = 0.601).

### 3.10 Malignant versus benign histology

The study included 121 (75%) patients with malignant histology (118 PTC and 3 medullary thyroid cancer) and 36 (23%) patients with benign pathology.

No significant differences in QOL parameters were found between the two groups.

## 4 DISCUSSION

The present study is the first to apply a comprehensive thyroid‐specific patient‐reported outcome tool to assess QOL with regard to the extent of thyroid surgery. Our results show that patients treated with total thyroidectomy had a worse self‐assessment score compared to patients treated with lobectomy, regardless of their tumor histological subtype or stage. Interestingly, when comparing patients receiving LT4 replacement therapy to euthyroid patients regardless of the extent of surgery, we found that hypothyroidism requiring LT4 therapy had a detrimental effect on QOL with regard to tiredness, emotional, and cosmetic scores as well as overall self‐assessment. In a subgroup analysis of lobectomy patients receiving LT4 therapy compared to lobectomy patients not receiving LT4 therapy, we found that tiredness score was worse in patients receiving LT4 therapy, implying that quality of life was determined by hypothyroidism rather than extent of neck surgery. Patients treated with lobectomy who developed hypothyroidism had similar quality of life to patients treated with total thyroidectomy, while patients treated with lobectomy and remained euthyroid had the best quality of life. Noteworthy, patients under TSH suppression also had worse overall self‐assessment score compared to the euthyroid group.

Previous studies have attempted to address the issue of QOL following thyroidectomy using non‐organ specific general self‐assessment tools. A study by Shah et al.^8^ tried to determine whether unilateral thyroid lobectomy has a less detrimental effect on QOL than total thyroidectomy and found that QOL is not significantly impacted by the extent of surgery and should not be a factor in the decision‐making process for the treatment of low‐risk WDTC, although patients with cancer had a greater drop in quality of life after 6 months compared to patients with benign disease. Interestingly, we did not see a significant impact on QOL in patients with malignant versus benign histology. In a study by Bongers et al.^9^ comparing long‐term quality of life in patients with low‐risk WDTC, QOL was not significantly different between patients treated with total thyroidectomy compared to unilateral thyroid lobectomy. In their survey, 40% of the patients responded more than 5 years after their operation. In a secondary analysis, concern about disease recurrence appeared to be higher in individuals treated with unilateral thyroid lobectomy. These findings correlate to ours as unilateral thyroid lobectomy patients did not have a better QOL compared to total thyroidectomy patients (except for the “self‐assessment” subcategory). Only when factoring in the hormonal balance we found major differences in QOL.

Previous studies trying to elicit the role of hypothyroidism or hyperthyroidism after thyroid surgery were mainly focused on TSH measurements. Hoftijzer et al.^10^ claimed that patients cured from WDTC have impaired QOL independent of TSH levels. They also found that QOL parameters may improve over time. Our results support the notion that TSH levels do not correspond with QOL when comparing patients with TSH suppression to patients with TSH levels within normal range but implicate the need for exogenous hormone to be detrimental to QOL.

Despite the general notion that patients on long‐term hormone replacement report persistent fatigue and impaired general well‐being, its impact on neuropsychological function has been studied only in a limited number of patients,^11^, ^12^ suggesting impaired cognitive functioning, especially on complex attention tasks and verbal memory tests.

The association between thyroid hormone deficiency, anxiety, and depression has been reported extensively.^13^ However, it was Saraven et al.^14^ in a community‐based study, who showed for the first time that patients on LT4 therapy and TSH levels within normal range have impaired psychological well‐being compared to a matched control group. This is in accordance with our findings of worse emotional score in patients receiving LT4 who have normal TSH levels. Our results as well as the work by Saravan et al. shines a spotlight on deficiencies that might lie beyond TSH levels, affecting QOL of patients.

Since most patients in this study are women, we sought to rule out menopause as a confounding factor that may affect QOL. When comparing women over and under 50 years of age we did not find any significant difference in QOL parameters between the two age groups.

Another treatment modality that may affect quality of life is adjuvant RAI treatment. Interestingly, we did not find significant differences in QOL between patients who received RAI and those who did not. Toxicity from RAI is well documented and consists most commonly from transient altered taste, and acute or chronic sialadenitis.^15^

Almeida et al.^16^ found that patients who received more than 150 mCi of RAI reported significantly worse pain, swallowing, chewing, speech, taste, anxiety, and composite scores. To evaluate how patients perceive their illness, Hirsch et al.^17^ studied 110 who completed an illness perception questionnaire. They demonstrated that patients with WDTC perceive their illness on a subjective, emotional basis unrelated to its actual severity. Specifically, the number of iodine treatments were the most predominant factor, significantly affecting three indices of negative disease perception: illness identity, severity of consequences, and emotional representation. Interestingly, number of surgeries were not correlated with illness perception.

Mendoza et al. concluded that patient care following thyroid cancer treatment could be improved by providing ongoing education about lifestyle factors related to cancer risk, disease surveillance, and resources for treating and coping with cancer.^18^

Our findings clearly indicate that QOL following thyroid surgery is significantly related to hypothyroidism and the requirement for LT4 therapy rather to the extent of surgery.

The current recommendations for treatment of hypothyroidism according to the 2013 European Thyroid Association guidelines advocate titrating levothyroxine replacement to TSH levels in the range of 0.4−4.0μIU/ml (desirable during LT4 titration to be in the lower half of the reference range −0.4 to 2.5 μIU/ml).^19^ Nevertheless, no single TSH concentration is likely to adequately reflect an euthyroid condition in every tissue implicating intrinsic imperfections of thyroxine replacement therapy.^20^ These imperfections might provide an explanation as to why LT4 therapy following thyroidectomy has a detrimental impact on QOL and be taken into consideration when deciding on the extent of surgery.

This is a retrospective study, non‐randomized, and the sample size is moderate but sufficient for statistical analysis.

In conclusion, QOL following thyroid surgery is significantly impacted by the requirement for LT4 therapy, rather to the extent of surgery. The best QOL was observed in patients following thyroid lobectomy who did not require LT4 therapy.

The possibility of requiring LT4 therapy following thyroidectomy and its implications should be openly discussed with the patient when counseling them on the extent of surgery.

## AUTHOR CONTRIBUTIONS

**Dan Yaniv**: analysis and interpretation of results and draft manuscript preparation. **Igor Vainer**: data collection and analysis and interpretation of results. **Ido Amir**: data collection. **Eyal Robenshtok**: review of results and approval of the final version of the manuscript. **Dania Hirsch**: review of results and approval of the final version of the manuscript. **Torquil Watt**: study conception and design. **Ohad Hilly**: review of results and approval of the final version of the manuscript. **Yotam Shkedy**: review of results and approval of the final version of the manuscript. **Thomas Shpitzer**: review of results and approval of the final version of the manuscript. **Gideon Bachar**: review of results and approval of the final version of the manuscript. **Raphael Feinmesser**: study conception and design, review of results, and approval of the final version of the manuscript. **Aviram Mizrachi**: study conception and design, review of results, and approval of the final version of the manuscript.

## CONFLICT OF INTEREST

The authors declare no conflict of interest.

## SYNOPSIS

Comparison between hemithyroidectomy and total thyroidectomy patients with thyroid‐specific ThyPRO QOL questionnaire shows quality of life following thyroid surgery is significantly related to hypothyroidism and the requirement for LT4 therapy.

## Data Availability

The data that support the findings of this study are available on request from the corresponding author. The data are not publicly available due to privacy or ethical restrictions.
